# Glomerular hyperfiltration may be a novel risk factor of restrictive spirometry pattern: Analysis of the Korea National Health and Nutrition Examination Survey (KNHANES) 2009-2015

**DOI:** 10.1371/journal.pone.0223050

**Published:** 2019-09-25

**Authors:** Hong Il Lim, Sang Jin Jun, Sung Woo Lee

**Affiliations:** Department of Internal Medicine, Nowon Eulji Medical Center, Eulji University, Seoul, Korea; Istituto Di Ricerche Farmacologiche Mario Negri, ITALY

## Abstract

**Background and objectives:**

There have been limited studies regarding the association between glomerular hyperfiltration (GHF) and restrictive spirometry pattern (RSP) in Korean adults.

**Methods:**

We used data of 23,189 adults from the Korea National Health and Nutritional Examination Survey 2009–2015 with a complete data set including spirometry, serum creatinine, and anthropometric measurements. Spirometry data included the forced expiratory volume in one second (FEV1) and forced vital capacity (FVC). We defined GHF as the >90th percentile of age & sex adjusted estimated glomerular filtration rate (eGFR), and RSP was defined as an FVC <80%-predicted value and an FEV1/FVC ratio ≥0.7.

**Results:**

Participants with RSP showed higher blood pressure, fasting glucose, and triglyceride, reduced high density lipoprotein cholesterol, and central obesity, which resulted in a higher prevalence of metabolic syndrome (MetS) compared to those without RSP. Multivariate logistic regression revealed that the odds for RSP were significantly increased with an increased number of MetS components. In addition, increased eGFR was associated with decreased FVC, showing an inverted J-shaped relationship in a multivariate generalized additive model analysis. In the multivariate logistic regression analysis, the adjusted odds ratio and 95% confidence interval of GHF for RSP was 1.184 (1.026–1.368, *P* = 0.021), which was evident in groups without metabolic disorders.

**Conclusions:**

We concluded that GHF was associated with increased odds for RSP, particularly in groups without metabolic disorders. Further prospective studies are needed to confirm our study results.

## Introduction

In ordinary practice, spirometry is commonly used to measure the pulmonary function [1]. Although airflow limitation is best reflected by a reduced forced expiratory volume in one second (FEV1) [2], a reduced forced vital capacity (FVC) may not necessarily be caused due to lung restriction [3, 4]. Therefore, the clinical interpretation of corresponding reductions in FVC and FEV1, leading to a preserved FEV1/FVC ratio, has to be deferred until a reduced lung volume is confirmed by volumetric measurements. However, this abnormal spirometry phenotype, which is now referred to as restrictive spirometry pattern (RSP) [5], has been consistently reported to be associated with an increased risk of insulin resistance (IR) [6, 7], metabolic syndrome (MetS) [8–11], diabetes [12–16], cardiovascular disease [6, 15, 17, 18], and all-cause death [11].This suggests that RSP may be a metabolic marker and not just a simple surrogate of lung restriction. To better characterize the RSP, its associated factors need to be explored.

Kidney function may be closely associated with RSP. The percentage of RSP increased with the progression of CKD [19], which may be partly attributed to fluid overload associated with a decreased kidney function [20]. However, Navaneethan et al. reported that a 5-ml/min/1.73 m^2^ decrease in estimated glomerular filtration rate (eGFR) resulted in decreased odds for RSP with an odds ratio (OR) and 95% confidence interval (CI) of 0.96 and 0.93–0.99, respectively, suggesting a positive relationship between eGFR and the risk of RSP, after evaluating data of 7,610 participants in the National Health and Nutrition Examination Survey (NHANES) 2007–2012 [21]. Since glomerular hyperfiltration (GHF) may be associated with MetS and IR [22], and RSP is closely related to MetS and IR, we hypothesized that GHF may be independently associated with an increased risk of RSP. To investigate this hypothesis, we first assessed the association between metabolic risk factors and RSP, and subsequently analyzed a potential relationship between GHF and RSP, using data from the Korea NHANES (KNHANES), a nationwide government-administered survey.

## Material and methods

### Participants

The KNHANES has been performed periodically since 1998 to assess the health and nutritional status of the civilian, non-institutionalized Korean population. Participants were selected using proportional-allocation systematic sampling with multistage stratification. The current study used data from the KNHANES 2009–2015. Of 73,639 candidates, 59,015 people agreed to participate in the KNHANES 2009–2015 (participation rate, 80.1%). The protocol comprised a health-questionnaire survey, a health examination, and a nutrition survey. Among 59,015 participants, we excluded 14,043 people aged <20 years. Of the remaining 44,972 adult participants, 20,912 and 871 additional subjects with missing spirometry and creatinine values, respectively, were excluded. Therefore, this study enrolled 23,189 participants.

### Ethics statement

The study protocol complied with the Declaration of Helsinki. Full approval of the study was obtained from the Institutional Review Board of the Korea Centers for Disease Control (IRB number: 2009-01CON-03-2C, 2010-02CON-21-C, 2011-02CON-06-C, 2012-01EXP-01-2C, 2013-07CON-03-4C, 2013-12EXP-03-5C). All data were fully anonymized before we accessed them. KNHANES participants provided informed written consent to have their data used in research.

### Spirometric measurements

As described in detail previously [23], spirometry was performed using a dry rolling seal spirometer (Model 2130; Sensor Medics, Yorba Linda, CA, USA). Trained clinical technicians obtained spirometry data including FEV1, FVC, and FEV1/FVC on site and transferred them to an internet review center for processing. The data were compared against criteria metrics for acceptability, reproducibility, and quality control by a principal investigator to validate the data and to store them in a Korea Centers for Disease Control repository management system, in accordance with the ATS/ERS recommendations [1]. Two criteria were applied to the spirometry data: 1) two or more acceptable spirometry curves to ensure correct inspiration and 6s expiration and 2) 150-ml inter-measurement variability in FVC and FEV1. The spirometry tests were undertaken without the use of a bronchodilator. Age-, sex-, and height-adjusted normal predicted values of FVC and FEV1 in the general Korean population were used to calculate the values of percent-predicted FVC and FEV1.

### Other measurements

As described in detail previously [24], a standardized interview was conducted in the homes of the participants to collect information regarding demographic variables, medical history, medications used, and a variety of other health-related variables. BP was measured three times in accordance with the standard protocol, and the mean values of these three measures were used as the representative BP. Height was measured in the erect position with a stadiometer (Seca 225; Seca, Germany) to the nearest 0.1 cm. Body weight was measured in a light gown with bare feet using a digital scale (GL-6000-20; G-tech, Korea) to the nearest 0.1 kg, and the body mass index was calculated by dividing the weight by the square of the height (kg/m^2^). Waist circumference (WC) was measured with a tape measure (Seca 200; Seca) to the nearest 0.1 cm at the midpoint between the lower border of the rib cage and the highest point of the iliac crest of the subject. Blood samples were collected in the morning after at least 8 h fasting and analyzed at a central laboratory (Neodin Medical Institute, Seoul, Korea). Values of HDL-C, TG, fasting glucose, and serum creatinine were measured by using a Hitachi Automatic Analyzer 7600 (Hitachi, Japan).

### Definitions

The RSP was defined as an FVC <80%-predicted value and an FEV1/FVC ratio ≥0.7 [5]. GHF was defined as all subjects as the >90th percentile in the distribution of residuals from a multiple linear regression analysis where we used the eGFR as a dependent variable and age and sex as independent variables [25]. Five components of a MetS were defined following the recommendations of the International Diabetes Federation [26]. First, raised BP was defined as a systolic BP ≥130 mm Hg, a diastolic BP ≥85 mmHg, treatment with anti-hypertensive drugs, or a previous diagnosis of hypertension by a physician. Second, raised fasting glucose was defined as a fasting glucose ≥100 mg/dl, treatment with insulin or oral anti-diabetic drugs, or a previous diagnosis of diabetes by a physician. Third, raised TG was defined as TG ≥150 mg/dl or treatment with anti-dyslipidemic drugs. Fourth, reduced HDL-C was defined as HDL-C <40 mg/dl in men and <50 mg/dl in women. Finally, central obesity was defined as WC ≥90 cm in men and ≥80 cm in women. MetS was defined as three or more out of the five MetS components [27]. Alcohol drinking was defined as drinking alcoholic beverages more than twice a week. A high monthly income was defined as the highest quartile of the monthly household income in Korea (≥4,730,000 KRW). Bronchial asthma, allergic rhinitis, atopic dermatitis, and pulmonary tuberculosis (past or current) were defined as a disease diagnosis by a physician. A previous cardiovascular (CV) disease was defined as the diagnosis of a cerebrovascular disease, angina, or myocardial infarction by a physician. The eGFR was calculated using the Chronic Kidney Disease Epidemiology Collaboration equation [28], which was frequently used to define GHF [29]. Proteinuria was defined as ≥1+ protein in a dipstick urinalysis.

### Statistical analysis

Continuous variables are expressed as the mean ± standard deviation and categorical variables as percentages. Differences were analyzed by Student’s t-test for continuous variables and a chi-squared test for categorical variables. The OR and 95% CI were determined using a logistic regression analysis. In the multivariate analysis, covariates were chosen based on clinical and statistical relevance. Using R Statistics (version 3.03), the locally weighted scatterplot smoothing (LOWESS) regression curve on a scatter plot between eGFR and percent-predicted FVC was used to assess the simple relationship between both variables. Subsequently, a multivariate generalized additive model (GAM) for Gaussian distribution [30] was adapted to visualize the associations between eGFR and percent-predicted FVC after adjusting for the same variables utilized in the multivariate logistic regression analysis. A *P*-value of <0.05 was considered statistically significant. Unless otherwise specified, all analyses were performed using SPSS Version 22 (released 2013, IBM Corp., Armonk, NY).

## Results

The mean age of the 23,189 participants was 55.3 ± 12.2 years and 44.5% were men. The percentages of participants with raised BP, raised fasting glucose, raised TG, reduced HDL-C, and central obesity were 37.7%, 37.3%, 33.2%, 41.8%, and 41.9%, respectively. The overall prevalence of MetS in the study was 32.9%. The mean eGFR was 90.8 ± 15.2 ml/min/1.73m^2^, and proteinuria was present in 1.1% of the study population. The mean FVC, FEV1, and FEV1/FVC were 92.8%-predicted, 92.4%-predicted, and 0.78, respectively. The RSP prevalence was 10.3% in the study population.

**Fig 1:**
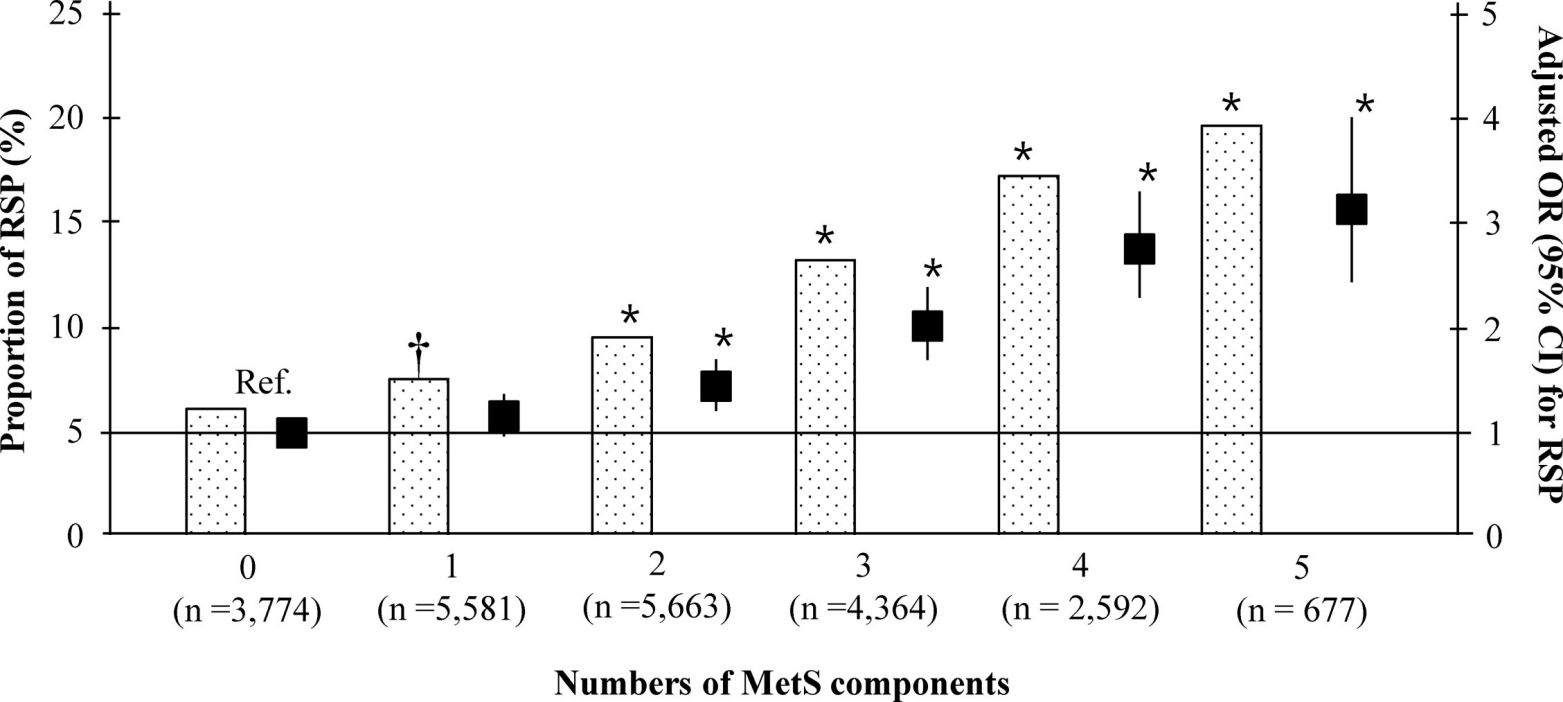
Association between MetS and RSP. MetS, metabolic syndrome; RSP, respiratory spirometry pattern; OR, odds ratio; CI, confidence interval; Ref. reference. Adjusted OR and 95% CI were analyzed using multivariate logistic regression analysis, entering into age, sex, status of smoking, alcohol drinking, education, income, and mental stress, pulmonary tuberculosis, bronchial asthma, allergic rhinitis, atopic dermatitis, previous cardiovascular disease, proteinuria, estimated glomerular filtration rate group, white blood cells, and hemoglobin as covariates. * *P* <0.001 and † *P* = 0.009, compared to the reference.

We compared the baseline characteristics of the study population according to their RSP status (Table 1). The mean age and the proportion of men were higher in the group with RSP than in that without RSP, whereas the rates of alcohol drinking, college graduates, and a high monthly income were lower. Although more people with RSP had pulmonary tuberculosis, the rate of allergic rhinitis was lower in this group than in that without RSP. White blood cell (WBC) levels were higher and previous CV disease was more prevalent in the RSP group compared to the group without RSP. Study participants with RSP presented higher prevalences of raised BP, raised fasting glucose levels, raised TG, reduced HDL-C, and central obesity, resulting in a higher MetS prevalence compared to participants without RSP. In the multivariate logistic regression analysis, increased systolic BP, WC, and fasting glucose, and decreased HDL-C, previous CV disease, and increased hemoglobin levels were independently associated with increased odds for RSP (Table 2). We also found that the odds for RSP were significantly increased with an increase in the number of MetS components (Fig 1).

**Table 1:** Clinical characteristics of the study population by the status of restrictive spirometry pattern.

	RSP negative (n = 20,791)	RSP positive (n = 2,398)	*P*
Age (years)	54.9 ± 12.2	58.5 ± 12.4	<0.001
Men, n (%)	9,169, (44.1)	1,159, (48.3)	<0.001
Current smoking, n (%)	3,955, (19.4)	452, (19.1)	0.769
Alcohol drinking, n (%)	4,627, (22.7)	493, (20.9)	0.042
College graduate, n (%)	4,935, (24.4)	491, (20.9)	<0.001
High monthly income, n (%)	5,257, (25.5)	491, (20.7)	<0.001
High mental stress, n (%)	4,841, (23.7)	533, (22.6)	0.210
Pulmonary TB, n (%)	1,000, (4.9)	176, (7.5)	<0.001
Bronchial asthma, n (%)	611, (3.0)	76, (3.2)	0.574
Allergic rhinitis, n (%)	2,142, (10.6)	182, (7.7)	<0.001
Atopic dermatitis, n (%)	305, (1.5)	35, (1.5)	0.940
Raised BP, n (%)	7,651, (36.8)	1,100, (45.9)	<0.001
Systolic BP (mm Hg)	122.0 ± 16.7	126.9 ± 17.7	<0.001
Diastolic BP (mm Hg)	77.6 ± 10.2	78.4 ± 10.7	0.001
Raised fasting glucose, n (%)	7,480, (36.5)	1,180, (49.6)	<0.001
Fasting glucose (mmol/l)	5.6 ± 1.3	5.9 ± 1.5	<0.001
Raised triglyceride, n (%)	6,744, (32.4)	955, (39.8)	<0.001
Triglyceride (mmol/l)	1.6 ± 1.2	1.8 ± 1.3	<0.001
Reduced HDL-C, n (%)	8,519, (41.4)	1,170, (49.4)	<0.001
HDL-C (mmol/l)	1.3 ± 0.3	1.2 ± 0.3	<0.001
Central obesity, n (%)	8,361, (40.2)	1,365, (57.0)	<0.001
Waist circumference (cm)	82.5 ± 8.9	86.3 ± 10.3	<0.001
BMI (kg/m^2^)	24.1 ± 3.0	25.3 ±3.7	<0.001
Metabolic syndrome, n (%)	6,478, (31.9)	1,155, (49.3)	<0.001
Previous CV disease, n (%)	796, (3.9)	173, (7.3)	<0.001
FVC (L)	3.6 ± 0.9	2.8 ± 0.7	<0.001
FVC (%-predicted)	95.0 ± 10.3	73.9 ± 6.0	<0.001
FEV1 (L)	2.8 ± 0.7	2.2 ± 0.5	<0.001
FEV1 (%-predicted)	94.1 ± 12.9	78.1 ± 8.3	<0.001
FEV1/FVC	0.78 ± 0.08	0.80 ± 0.06	<0.001
WBC (×10^3^/μl)	6.03 ± 1.70	6.31 ± 1.76	<0.001
Hemoglobin (g/dl)	14.0 ± 1.5	14.0 ± 1.6	0.405
GHF, n (%)	2,031, (9.8)	284 (11.8)	0.001
eGFR (ml/min/1.73m^2^)	91.1 ± 14.9	87.7 ± 16.6	<0.001
Proteinuria, n (%)	218, (1.1)	48, (2.1)	<0.001

RSP, respiratory spirometry pattern; TB, tuberculosis; BP, blood pressure; HDL-C, high density lipoprotein cholesterol; BMI, body mass index; CV, cardiovascular; FVC, forced vital capacity; FEV1, forced expiratory volume in 1 second; WBC, white blood cells; eGFR, estimated glomerular filtration rate; GHF, glomerular hyperfiltration. Values are expressed as mean ± standard deviation for continuous variables. Difference was analyzed by Student t-test for continuous variables and a chi-square test for categorical variables.

**Table 2:** Factors associated with restrictive spirometry pattern.

	Adjusted OR (95% CI)	*P*
Age (per 1 year increase)	1.011 (1.007–1.016)	<0.001
Men (vs. women)	1.077 (0.945–1.228)	0.267
GHF (yes vs. no)	1.184 (1.026–1.368)	0.021
Pulmonary TB (yes vs. no)	1.564 (1.311–1.867)	<0.001
Bronchial asthma (yes vs. no)	0.944 (0.732–1.217)	0.656
Allergic rhinitis (yes vs. no)	0.839 (0.709–0.992)	0.040
Atopic dermatitis (yes vs. no)	1.042 (0.714–1.520)	0.831
Previous CV disease (yes vs. no)	1.385 (1.153–1.662)	<0.001
SBP (per 1 mmHg increase)	1.011 (1.007–1.015)	<0.001
DBP (per 1 mmHg increase)	0.994 (0.988–1.001)	0.074
WC (per 1cm increase)	1.035 (1.029–1.041)	<0.001
Fasting glucose (per 1 mmol/l increase)	1.074 (1.041–1.107)	<0.001
HDL-C (per 1 mmol/l increase)	0.745 (0.624–0.889)	0.001
Triglyceride (per 1 mmol/l increase)	1.027 (0.991–1.065)	0.147
WBC (per 1000/μl increase)	1.027 (0.991–1.065)	0.147
Hemoglobin (per 1 g/dl increase)	1.041 (1.013–1.070)	0.004
Proteinuria (yes vs. no)	1.042 (0.714–1.520)	0.831

OR, odds ratio; CI, confidence interval; GHF, glomerular hyperfiltration; TB, tuberculosis; CV, cardiovascular; SBP, systolic blood pressure; DBP, diastolic blood pressure; WC, waist circumference; HDL-C, high density lipoprotein cholesterol; TG, triglyceride; WBC, white blood cells. Adjusted OR and 95% CI were calculated using multivariate logistic regression analysis. Status of alcohol drinking, smoking, education, income, and mental stress and all above variables were used as covariates.

**Fig 2:**
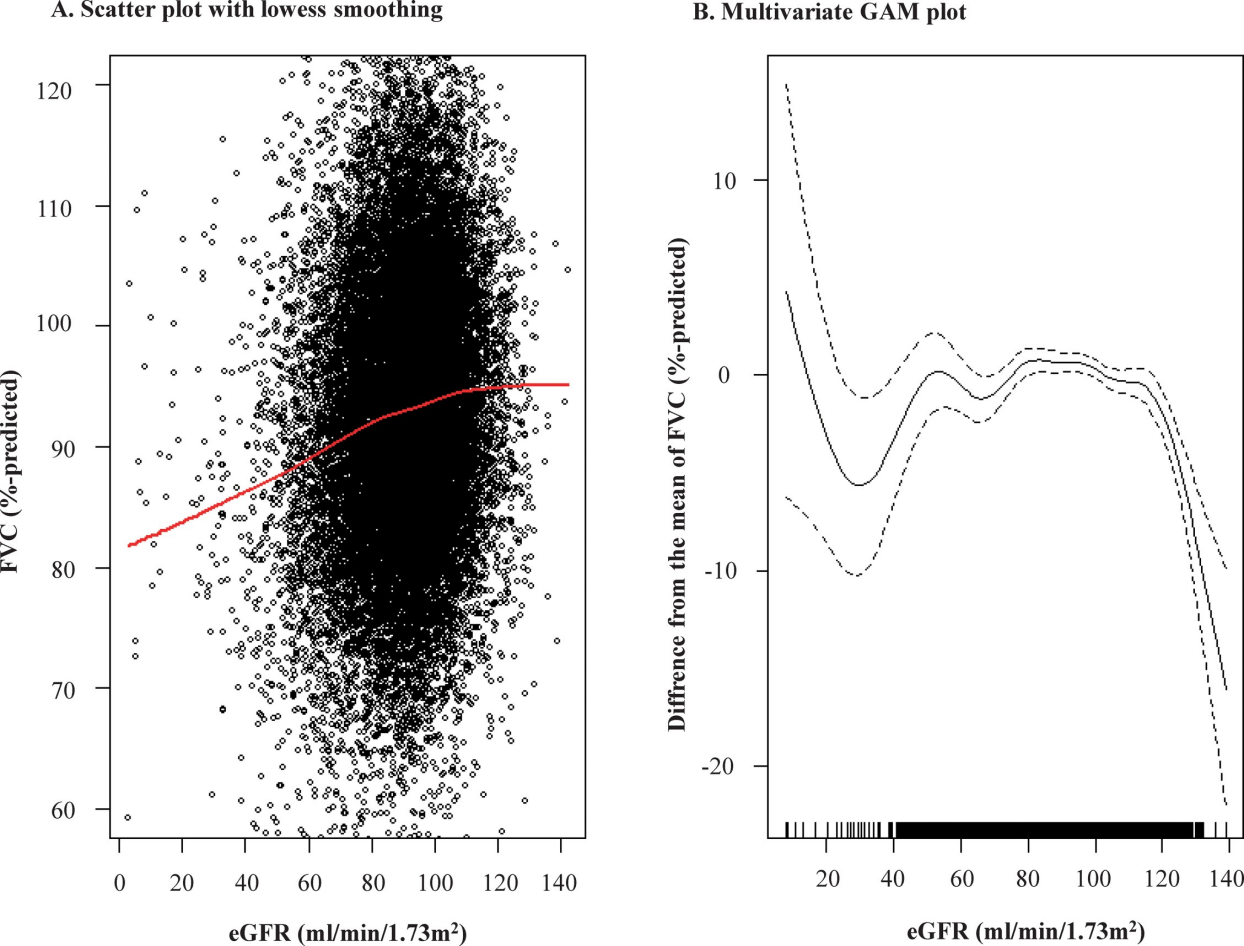
Relationship between kidney function and FVC. eGFR, estimated glomerular filtration rate; FVC, forced vital capacity; LOWESS, locally weighted scatterplot smoothing; GAM, generalized additive model. Panel A demonstrated the univariate relationship between eGFR and percent-predicted FVC. Upper (≥120%-predicted) and lower (<60%-predicted) one percent of FVC was truncated. The red line indicated in the scatter plot in the LOWESS regression curve between eGFR and percent-predicted FVC. Panel B demonstrated the multivariate relationship between eGFR and percent-predicted FVC using GAM analysis, entering into variables in Table 2 as covariates.

**Fig 3:**
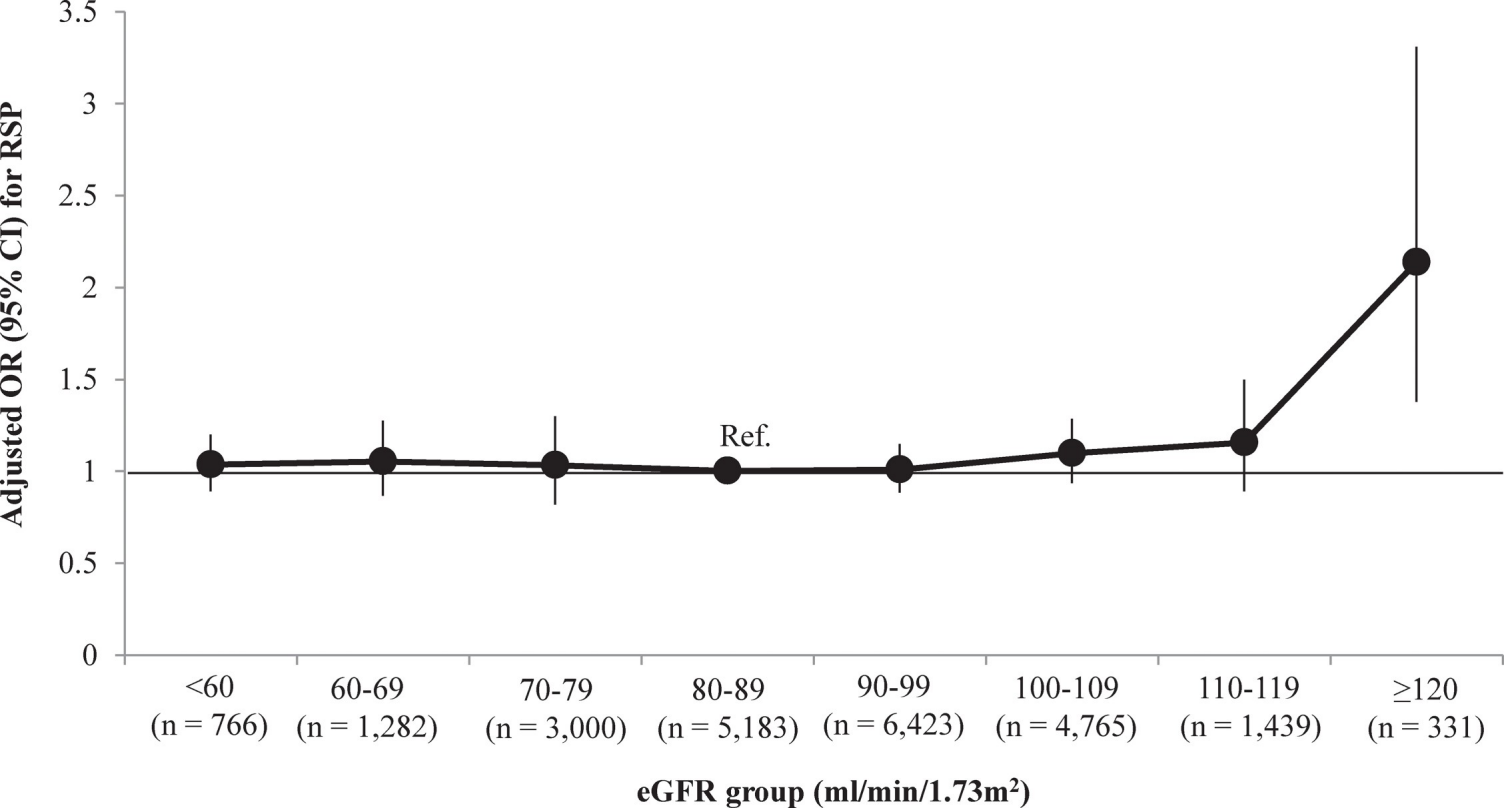
Association between eGFR group and RSP. RSP, respiratory spirometry pattern; eGFR, estimated glomerular filtration rate; OR, odds ratio; CI, confidence interval; Ref, reference. Adjusted OR and 95% CI were calculated using multivariate logistic regression analysis entering into variables in Table 2 as covariates.

Furthermore, we explored the relationship between eGFR and FVC. In a scatter plot, the LOWESS regression curve revealed that an increased eGFR was associated with an increased FVC, with a plateau for higher eGFR values (Fig 2A). However, in a multivariate GAM plot, adjusted for age, sex, status of smoking, alcohol drinking, education, income, mental stress, bronchial asthma, allergic rhinitis, atopic dermatitis, pulmonary tuberculosis, previous CV disease, proteinuria, systolic and diastolic BP, fasting glucose, TG, HDL-C, WC, WBC, and hemoglobin as covariates, an increased eGFR was significantly associated with a decreased FVC, particularly when the eGFR was higher than 120 ml/min/1.73m^2^ (Fig 2B). We also found in a multivariate logistic regression analysis that the OR (95% CI) of the eGFR ≥120 ml/min/1.73m^2^ group for RSP was 2.137 (1.379–3.310, *P* = 0.001) compared to the eGFR 80–89 ml/min/1.73m^2^ group (Fig 3), and GHF was independently associated with increased odds of RSP with OR (95% CI) of 1.184 (1.026–1.368, *P* = 0.021, Table 2).

**Fig 4:**
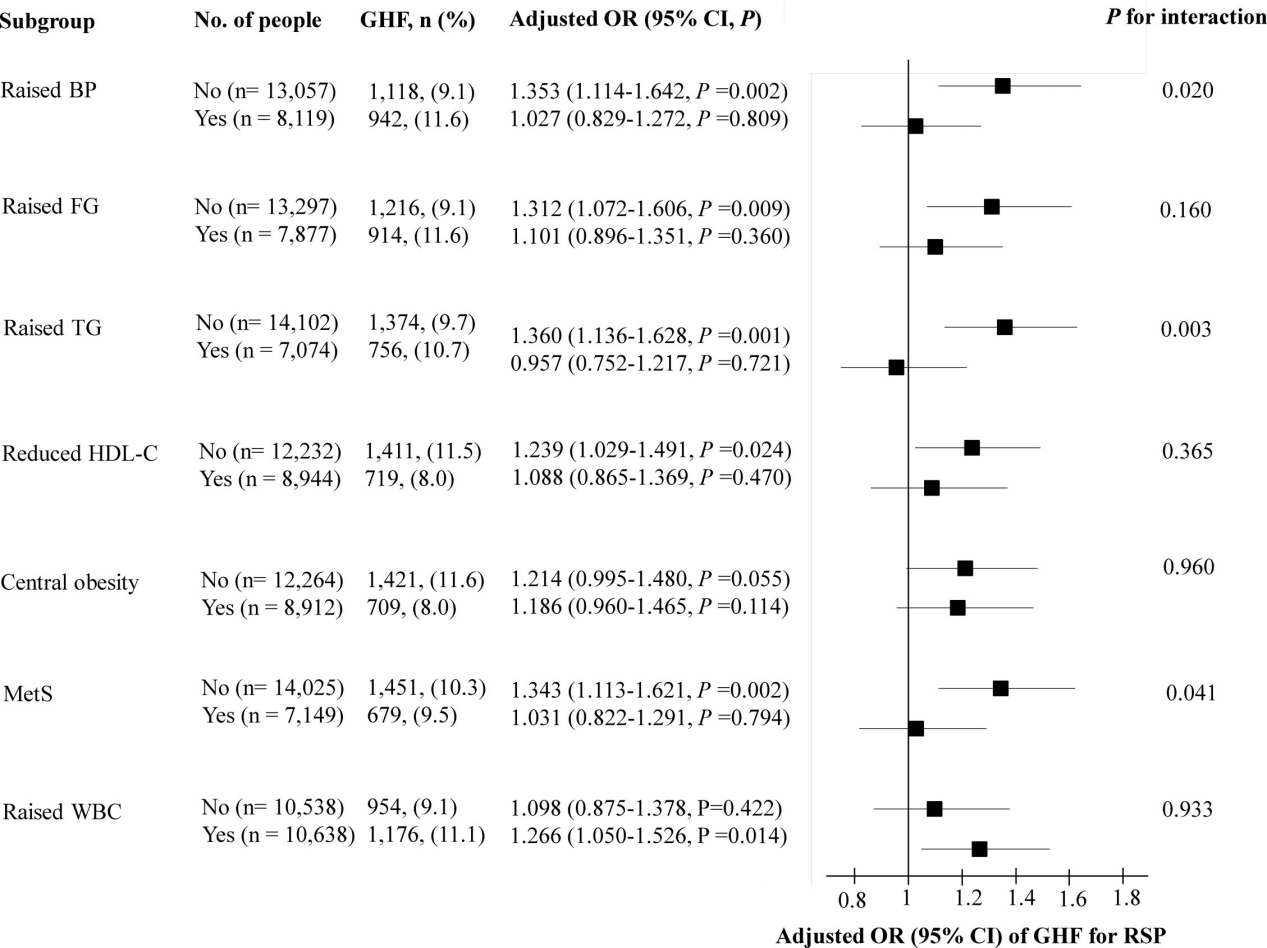
Subgroup analysis for the relationship between eGFR group and RSP. RSP, respiratory spirometry pattern; eGFR, estimated glomerular filtration rate; OR, odds ratio; CI, confidence interval; BP, blood pressure; FG, fasting glucose; TG, triglyceride; HDL-C, high density lipoprotein cholesterol; MetS, metabolic syndrome; WBC, white blood cells. Adjusted OR and 95% CI were analyzed using multivariate logistic regression analysis, entering into variables in Table 2 as covariates. Raised WBC was defined as above median of WBC levels (≥5820/μl). When covariates were chosen as subgroup, they were excluded from the model.

In a subgroup analysis, the odds of GHF for RSP were statistically significant only in subgroups without raised BP, raised fasting glucose, raised TG, reduced HDL-C, central obesity, MetS, but with raised WBC (Fig 4).

## Discussion

The clinical interpretation of a reduced FVC with preserved FEV1/FVC should be postponed until a volumetric method confirms a lung restriction because spirometry indicative of a restrictive lung disease does not always signify it [3, 4]. However, numerous studies suggest that this abnormal spirometry phenotype termed as RSP may be linked to metabolic disorders, including IR [6, 7], MetS [8–11], and diabetes [12–16]. The clinical characteristics of RSP beyond lung function should, therefore, be studied further; however, the potential relationship between RSP and metabolic disorders has rarely been evaluated in Asian populations [9, 11]. Hypothetically, lung function is closely related to kidney functions [19–21]. However, the association between kidney and lung functions has been studied infrequently, particularly in large-scale populations [21]. Therefore, we performed the current study and confirmed that metabolic disorders were independently associated with RSP in community-dwelling Korean adults. Furthermore, our study is the first description that GHF assessed by creatinine based eGFR was associated with increased odds for RSP.

In this study, we found that increased BP, WC, fasting glucose, and decreased HDL-C were independently associated with RSP. We also demonstrated that the odds for RSP were significantly greater with an increased presence of MetS components. These findings are in accordance with previous studies [8–11]. Analyzing data from 121,965 citizens of Paris, Leone et al. reported the RSP adjusted ORs (95% CI) of 1.18 (1.10–1.26), 1.29 (1.21–1.38), and 2.13 (1.96–2.32) for abnormalities in lipids, glucose, BP, and abdominal obesity, respectively [10]. Nakajima et al. observed in a study of 2,396 Japanese patients that the percent-predicted FVC was significantly decreased with an increase in the number of MetS components [9]. To date, however, the exact reason for these potential metabolic risks in RSP has not been fully explained yet. However, a close relationship between RSP and inflammation has been strongly hypothesized. C-reactive protein (CRP) is independently associated with a low FVC [6, 9, 11, 16]. Other inflammatory markers including interleukin-6, fibrinogen, alpha1-antitrypsin, haptoglobin, ceruloplasmin, and orosomucoid were also inversely associated with FVC [6, 16, 31]. Therefore, the role of systemic inflammation on the metabolic hazard of RSP should be analyzed further in future studies.

In our study, we identified that kidney function was independently associated with RSP, but the relationship was not simple. The LOWESS regression curve of the relationship between eGFR and FVC revealed that an increased eGFR was associated with an increased FVC, although a plateau was reached at very high eGFR values. Consistent with this finding, a 10- ml/min/1.73m^2^ increase in eGFR was associated with a 1.00%-predicted increased FVC (95% CI, 0.90%-1.10%; *P* <0.001) in the univariate linear regression analysis. However, the relationship does not seem to be linear because the multivariate approach attenuated the statistical significance: beta -0.056 (95% CI, -0.19–0.08, *P* = 0.407). To identify the potentially non-linear relationship, we performed a multivariate GAM plot. The eGFR displayed an inverted J-shaped association with FVC, meaning that an increased eGFR was significantly associated with a decreased FVC, particularly when the eGFR was sufficiently high. This was confirmed by the finding that people with GHF showed 18.4% higher odds for the presence of RSP than those without GHF.

It is well known that GHF is an independent predictor for the progression of CKD [32–35] and all-cause mortality [36]. Although several definitions of GHF including an eGFR above two standard deviations from the mean value have been utilized [29, 37], the influence of age and sex should be adjusted [25]. In this study, therefore, we defined GHF using age and sex adjusted eGFR. Although pregnancy, high protein diet, and hydration status can cause GHF [38–40], the most common conditions associated with GHF are metabolic disorders including diabetes [41], obesity [42], and MetS [43]. In this study, increased systolic BP, fasting glucose, and TG were independently associated with a GHF (S1 Table). Therefore, metabolic disorders may affect the relationship between GHF and RSP. In our subgroup analysis, however, an increased odds of GHF for RSP was best observed in groups without metabolic disorders, suggesting that other pathophysiological mechanisms may also underlie the relationship between GHF and RSP. Although controversial, there have been some studies suggesting the relationship between inflammation and GHF [44, 45]. In addition, our subgroup analysis revealed that the relationship between GHF and RSP was valid only in the group with a raised WBC. Therefore, systemic inflammation may affect the relationship between GHF and RSP.

A common feature among MetS, inflammation, GHF, and RSP is the lack of physical fitness and muscle strength. Decreased physical fitness and muscle strength are associated with an increased risk of MetS [46, 47]. They are also associated with increased systemic inflammation [48, 49]. Furthermore, the OR (95% CI) of physical inactivity for RSP was 8.1 (1.43–46.4) [50]. Decreased muscle mass is associated with low serum creatinine [51], causing a falsely high eGFR. It is therefore likely that the association between GHF and RSP at least in part is confounded by creatinine based eGFR. Studies using measured GFR are needed to clarify the association between MetS, GHF and RSP. In addition, the impact of physical fitness and muscle strength as a fundamental background on the relationship between MetS, GHF, and RSP should be studied further in future studies.

The present study has several limitations. Apart from using creatinine based eGFR, this was a cross-sectional study, and a causal relationship should be cautiously interpreted. Since the purpose of this study was to investigate the nature of the RSP, we defined the RSP as the outcome with MetS and GHF as the exposure variable. However, it is also true that RSP is an important exposure for metabolic disorders and kidney diseases [52, 53]. Therefore, multi-dimensional approaches are required for a better understanding of the RSP characteristics. Second, the RSP definition is still evolving, which limits the comparability among the published studies [5]. The consistent association between RSP and adverse outcomes may compensate this weakness to some extent. Third, the study population consisted of community-dwelling adults. Therefore, not many participants presented with CKD; 3.3% showed a decreased kidney function (eGFR <60 ml/min/1.73m^2^), while the rate of advanced CKD (eGFR <30ml/min/1.73m^2^) was only 0.2%. For this reason, the effect of a severely decreased eGFR on RSP could not be tested in this population and should be properly evaluated in a separate CKD population. Fourth, more advanced inflammation markers such as CRP, markers for IR, and muscle mass were not consistently evaluated in the KNHANES 2009–2015. Finally, the homogenous ethnicity of the patients limits the generalizability of this study.

In conclusion, metabolic disorders were independently associated with RSP in community dwelling Korean adults. In addition, GHF was associated with increased odds for RSP, particularly in groups without metabolic disorders. Further studies are required to characterize the nature of RSP in more detail, particularly focusing on the crosstalk between GHF, MetS, inflammation, physical fitness/muscle strength, and RSP.

## Supporting information

S1 Table Factors associated with glomerular hyperfiltration. (DOCX)

S1 Dataset Dataset for this study. (XLSX)
