# Supplementary material for: Glomerular hyperfiltration may be a novel risk factor of restrictive spirometry pattern: Analysis of the Korea National Health and Nutrition Examination Survey (KNHANES) 2009-2015
Source: PLoS One. 2019 Sep 25;14(9):e0223050. doi: 10.1371/journal.pone.0223050 (PMC6760802; doi:10.1371/journal.pone.0223050)
Supplement: S1 Table — (DOCX) [file pone.0223050.s001.docx]

**Supplemental Materials**

S1 Table. Factors associated with glomerular hyperfiltration

|  | **Univariate** | | **Multivariate** | |
| --- | --- | --- | --- | --- |
|  | OR (95% CI) | *P* | OR (95% CI) | *P* |
| Age (year)* | 1.017 (1.013-1.020) | <0.001 | 1.008 (1.003-1.013) | 0.001 |
| Men (vs. women) | 3.723 (3.384-4.095) | <0.001 | 4.884 (4.252-5.61) | <0.001 |
| Current smoking (yes vs. no) | 2.227 (2.025-2.448) | <0.001 | 1.294 (1.151-1.454) | <0.001 |
| Alcohol drinking (yes vs. no) | 2.505 (2.288-2.744) | <0.001 | 1.424 (1.276-1.588) | <0.001 |
| College graduate (yes vs. no) | 0.690 (0.617-0.771) | <0.001 | 0.678 (0.598-0.769) | <0.001 |
| High monthly income (yes vs. no) | 0.673 (0.604-0.751) | <0.001 | 0.800 (0.709-0.903) | <0.001 |
| High mental stress (yes vs. no) | 0.894 (0.805-0.992) | 0.035 | 1.016 (0.907-1.138) | 0.784 |
| **Systolic BP (mm Hg)*** | **1.010 (1.007-1.012)** | **<0.001** | **1.006 (1.002-1.01)** | **0.003** |
| **Diastolic BP (mm Hg)*** | **1.005 (1.001-1.010)** | **0.011** | 0.992 (0.985-0.998) | 0.014 |
| **Waist circumference (cm)*** | **1.009 (1.005-1.014)** | **<0.001** | 0.985 (0.979-0.991) | <0.001 |
| **Fasting glucose (mmol/l)*** | **1.130 (1.101-1.160)** | **<0.001** | **1.071 (1.036-1.107)** | **<0.001** |
| HDL-C (mmol/l)* | 0.936 (0.811-1.080) | 0.365 | 1.518 (1.281-1.799) | <0.001 |
| **Triglyceride (mmol/l)*** | **1.137 (1.106-1.168)** | **<0.001** | **1.088 (1.053-1.124)** | **<0.001** |
| Pulmonary TB (yes vs. no) | 1.414 (1.188-1.682) | <0.001 | 1.148 (0.953-1.384) | 0.147 |
| Bronchial asthma (yes vs. no) | 1.054 (0.823-1.351) | 0.677 | 1.242 (0.952-1.621) | 0.111 |
| Allergic rhinitis (yes vs. no) | 0.622 (0.525-0.736) | <0.001 | 0.738 (0.615-0.886) | 0.001 |
| Atopic dermatitis (yes vs. no) | 0.649 (0.424-0.993) | 0.046 | 0.699 (0.443-1.104) | 0.125 |
| Previous CV disease (yes vs. no) | 0.825 (0.655-1.038) | 0.101 | 0.629 (0.491-0.806) | <0.001 |
| Proteinuria (yes vs. no) | 1.044 (0.704-1.547) | 0.832 | 0.663 (0.435-1.01) | 0.056 |
| WBC (1000/μl)* | 1.090 (1.065-1.116) | <0.001 | 1.018 (0.99-1.047) | 0.217 |
| Hemoglobin (g/dl)* | 1.168 (1.136-1.202) | <0.001 | 0.841 (0.808-0.875) | <0.001 |

eGFR, estimated glomerular filtration rate; OR, odds ratio; CI, confidence interval; BP, blood pressure; HDL-C, high-density lipoprotein cholesterol; TB, tuberculosis; CV, cardiovascular; WBC, white blood cells. OR and 95% CI were analyzed using logistic regression analysis. In multivariate analysis, all above variables were entered into as covariates. * per 1 unit increase.
